# Experiences, mental well‐being and community‐based care needs of fathers of late preterm infants: A mixed‐methods pilot study

**DOI:** 10.1002/nop2.370

**Published:** 2019-11-06

**Authors:** Shahirose Sadrudin Premji, Sandra Reilly, Genevieve Currie, Aliyah Dosani, Lynnette May Oliver, Abhay K. Lodha, Marilyn Young, Marc Hall, Tyler Williamson

**Affiliations:** ^1^ School of Nursing Faculty of Health York University Toronto ON Canada; ^2^ Faculty of Nursing University of Calgary Calgary AB Canada; ^3^ School of Nursing and Midwifery Health, Community & Education, Mount Royal University Calgary AB Canada; ^4^ Department of Community Health Sciences Cumming School of Medicine University of Calgary Calgary AB Canada; ^5^ Division of Neonatology Department of Paediatrics Alberta Health Services Foothills Medical Centre Calgary AB Canada; ^6^ Prenatal & Postpartum Services Public Health Calgary Zone Alberta Health Services Calgary AB Canada

**Keywords:** community health nursing, fathers, infants, nurses, postpartum period, premature, public health, public health nursing

## Abstract

**Aims:**

We explore fathers' experience of caring for a late preterm infant including their stressors, needs and corresponding interventions proffered by public health nurses.

**Design:**

Pilot mixed‐methods exploratory sequential design.

**Methods:**

We collected (a) qualitative data from semi‐structured interviews (*N = *5) and (b) quantitative data (*N = *31) about fathers' levels of stress (Parenting Stress Index), anxiety (Speilberger State–Trait Anxiety) and depression (Edinburgh Postnatal Depression Scale) at 6–8 weeks after birth of their infant.

**Results:**

Fathers appreciated their infant was born ‘early’, however, discovered through experience the demands of their infant, which appeared as stress (child and parent domains) and anxiety. Themes: hypervigilance in care explained the fathers' sense of competency and role restriction; infant fatigue and parental feeding elucidated the stressful aspect of father–infant interaction. Unscientific advice from healthcare providers was confusing and frustrating while uncertainty of rehospitalization caused worries, fears or stress. One father experienced depressive symptoms.

## 1 INTRODUCTION

In Canada, Alberta has the highest provincial rate of in‐hospital preterm birth (8.3% by hospital reporting) (Canadian Institute of Health Informatics, 2013) and largest proportion of early maternal discharge from hospital following delivery, both vaginal and caesarean (Public Health Agency of Canada, 2008). Late preterm infants (LPIs), 34 0/7 to 36 6/7 weeks' gestational age, represent a majority and a distinct subpopulation of preterm infants (Blondel et al., 2002; Engle, Tomashek, & Wallman, 2007). Compared with full‐term infants, they present with (a) greater morbidities at birth (MacBird et al., 2010), (b) greater neonatal and infant mortality (Khashu, Narayanan, Bhargava, & Osiovich, 2009) and (c) higher hospital readmission rates especially in the first week of life (McLaurin, Hall, Jackson, Owens, & Mahadevia, 2009). Nevertheless, data do not indicate a statistically significant difference in length of stay in birth hospital (in days) compared with term infants, when considering mode of delivery (Wang, Dorer, Fleming, & Catlin, 2004).

Public health nurses (PHNs) in Alberta support LPIs and their families following discharge from the birth hospital. With limited research to guide them in delivering care of LPIs in the home or clinic, PHNs adapt care practices for full‐term infants (Premji, Young, Rogers, & Reilly, 2012). When worried, families often become dependent on emergency departments or primary care practitioners for morbidities such as feeding difficulties (Jain & Cheng, 2006), which can increase parental stress (Leigh & Milgrom, 2008), distress (Tu et al., 2007) and anxiety (Hummel, 2003). Parents also demonstrate an increased risk of postpartum depression (PPD) at 3 months (corrected age) compared with parents of full‐term infants, regardless of the severity of their infants' illnesses (Mehler et al., 2014).

Mothers and fathers react differently to parenting a preterm infant (Howe, Sheu, Wang, & Hsu, 2014) suggesting a gendered nature of parenthood. Whereas fathers experience higher overall stress, mothers report social isolation and lack of support from their partners (Howe et al., 2014). Fathers' concerns centre on the well‐being of the mother, whereas mothers worry about their baby (Hagen, Iversen, & Svindseth, 2016). Regarding building a relationship with the infant, mothers related the need to regain the relationship lost because of a premature birth and hospitalization while fathers related the desire to begin a new relationship (Fegran, Helseth, & Fagermoen, 2008). In contrast to mothers, who experience the ‘immediate postnatal period as surreal and strange’, fathers are immediately ready to be involved in care following the birth of their premature infant (Fegran et al., 2008). Fathers, however, pursued balance between parenting role and work commitments (Jackson, Ternestedt, & Schollin, 2003) and had lower parenting self‐efficacy than mothers at 3 months following the birth of their infant (Feeley, Gottlieb, & Zelkowitz, 2007). Fathers' lack of engagement can interfere with child development (Sarkadi, Kristiansson, Oberklaid, & Bremberg, 2008; Wong et al., 2016) and have long‐term consequences for the LPIs, such as physical, cognitive, social and emotional delays (McGowan, Alderdice, Holmes, & Johnston, 2011). Studies narrated above examine parent's experience of caring for either preterm infant in general or very low‐birthweight infants or are situated in the neonatal intensive care units; hence, they fail to capture the unique experiences of fathers caring for LPIs in the community.

## 2 MODELS GUIDING THE RESEARCH QUESTIONS

This study, which was designed as part of a larger study focusing on public health nurses and mothers, aimed to describe fathers' experiences of caring for LPIs in the first 2 months following discharge to guide public health nursing interventions for fathers. Abidin (1995) and Leigh and Milgrom (2008) provided a useful frame of reference for our research questions as they emphasize parental stress/anxiety and relate it to depression. The nature of the relationship between parenting stress and postpartum depression is reciprocal (Leigh & Milgrom, 2008). Moreover, paternal postpartum depression can have an enduring impact on the mother's mental health (Goodman, 2004), family dynamics (Goodman, 2004; Tammentie, Tarkka, Astedt‐Kurki, Paavilainen, & Laippala, 2004), father–infant interaction and all dimension of child development (i.e. physical, emotional, cognitive, language, social and attention) (Sethna, Murray, Netsi, Psychogiou, & Ramchandani, 2015). Abidin's model represents parenting stress as an imbalance between the needs associated with caring for the infant and the perceived resources available to meet these needs (Abidin, 1995; Misri, Reebye, Milis, & Shah, 2006). This model focuses on parenting stress, specifically the parent's characteristics, child's characteristics and situational factors (Abidin, 1995). Leigh and Milgrom's psychosocial model, on the other hand, focuses on adjustment difficulties related to parenthood, which present early as a result of excessive parental anxiety, poor coping and lack of confidence in care. Excessive parental anxiety contributes to early parenting stress (Abidin, 1995; Leigh & Milgrom, 2008). This study therefore asked the following research questions: (a) what does it mean to be a father of a LPI?; (b) what are the perceived stressors and level of stress experienced by fathers of LPIs?; and (c) what is the rate of postpartum depressive symptoms in a sample of fathers of LPIs?

## 3 METHODS

### 3.1 Study design

We used a mixed‐methods exploratory sequential design for an in‐depth examination about the complexities of caring for the ordinary and special needs of LPIs (i.e. morbidities). We aimed to complete a small well‐planned pilot study to determine feasibility of a large‐scale study. First, a descriptive phenomenological inquiry elicited the fathers' experience (*N = *5) of caring for their LPI and permitted us to capture the complexity and diversity of challenges faced in the provision of such care. We also gathered quantitative data about stress, anxiety and depression by means of a descriptive survey with a convenience sample of fathers (*N = *31).

### 3.2 Sample of fathers and recruitment

**Figure 1:**
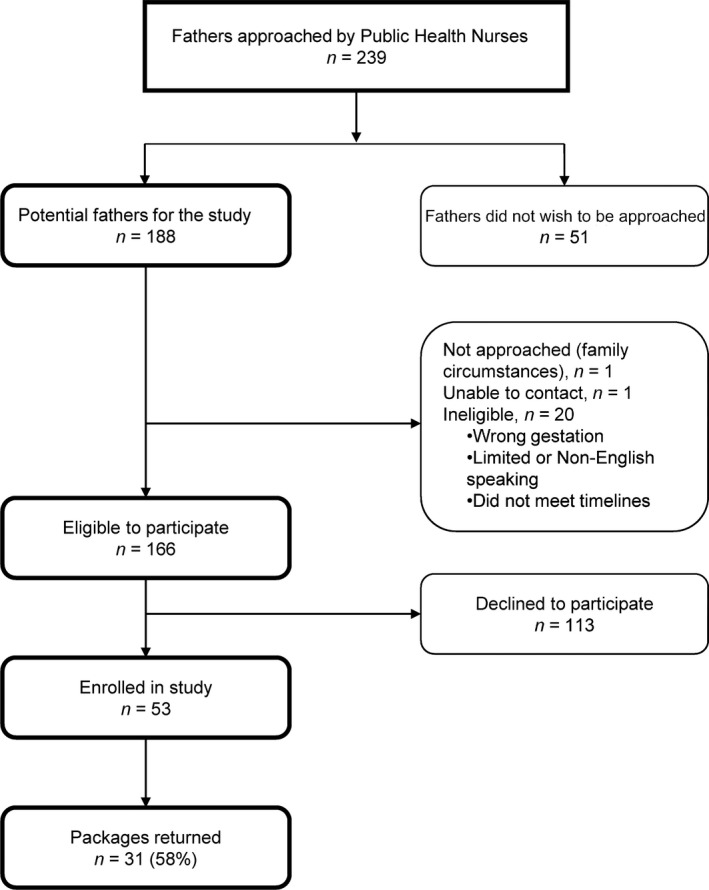
Flow through the study

All English‐literate fathers of LPIs, regardless of mode of delivery, admission status of the infant, or length of stay, were eligible to participate. Between April 2013 and June 2014, PHNs approached all potentially eligible families (*N = *239), provided an overview of the study and, if acceptable to the families (*N = *188, 79%), secured permission for further contact from the research team (see Figure 1 for study flow). Mothers eligible for the study (*N = *166) indicated father's willingness to participate (*N = *53), lack thereof (*N = *113), or explained that the father was ‘not around’ (*N = *1) or out of the country (*N = *1). Most of the fathers (*N* = 48) gave permission to be contacted for an interview. Fathers were identified for an interview through purposive sampling based on selection of fathers whose infants were within 1 month of age or between one and 2 months of age. Our intent was to explore fathers' needs and provision of care provided by public health nurses with LPIs zero to 2 months of age in Calgary, Alberta. Recruiting fathers proved challenging as despite contacting fifteen fathers only five agreed to be interviewed and 10 did not return our calls. Thirty‐one of the 53 fathers (58%) completed the survey at 6–8 weeks postpartum. To facilitate a high response rate, we used a telephone reminder with the mothers at 6–8 weeks, a postcard thanking participants and an unconditional incentive of a $20 gift certificate for groceries (Rosoff et al., 2005).

### 3.3 Data sources and main measurements

Five fathers agreed to in‐depth, face‐to‐face, semi‐structured recorded interviews, 60–90 min in duration undertaken in their home as requested. The conceptual models of Abidin (1995) and Leigh and Milgrom (2008) guided data collection. Abidin's model represents parenting stress as an imbalance between available resources and current demands of care (Abidin, 1995; Misri et al., 2006). Factors contributing to parenting stress include parents' and child's characteristics and situational factors (Wong et al., 2016). Leigh and Milgrom's (2008) psychosocial model emphasizes the reciprocal relationship between parenting stress and PPD.

The Parenting Stress Index (PSI), a 101‐item self‐report, assesses paternal stress in terms of raw scores for total stress (reliability coefficient .95), child and parent domain stress (reliability coefficient .89 and .93, respectively) and life stress; scores at or above the 85th percentile indicate high stress (Abidin, 1995; Loyd & Abidin, 1985). The PSI has high degree of internal consistency (i.e. reliable), is stable over time and has high factor validity suggesting that information can be gleaned with respect to the contribution of each of the domains to overall stress (Loyd & Abidin, 1985). Fathers' raw scores and T‐Scores facilitate comparison between child and parent domains (Abidin, 1995). The modified 40‐item Spielberger State–Trait Anxiety Inventory (STAI) Form Y appraises temporary or emotional state anxiety (test–retest correlations .54) and personality trait anxiety (test–retest correlation .86); elevated scores from 20–80 indicate high anxiety (Spielberger, Gorsuch, Lushene, Vagg, & Jacobs, 1983). Typical scores for people with diagnosed anxiety fall in the range 47–61 (McDowell, 2006), which provided a cut‐off for our sample. The modified STAI offers many advantages as it distinguishes temporary or emotional state anxiety from long‐standing personality trait anxiety and factor analysis determined that it is stable and more consistent than the original STAI scale (Spielberger & Vagg, 1984). The Edinburg Postnatal Depression Scale (EPDS), used to assess parental depressive symptoms (not diagnose depression), includes 10 items and has an optimum cut‐off of >9 or 10, which demarcates fathers as depressed or non‐depressed (Edmondson, Psychogiou, Vlachos, Netsi, & Ramchandani, 2010; Matthey, Barnett, Kavanagh, & Howie, 2001). The EPDS has a sensitivity and specificity of 89.5% and 78.2%, respectively (Edmondson et al., 2010), internal consistency of .81 and split‐half reliability of .78 (Matthey et al., 2001).

Data were collected through a mailed self‐administered questionnaire, sent only to fathers who gave consent, that elicited information about the characteristics of fathers (see Table 1). The completed questionnaire was returned using the envelope (addressed and stamped) included in the package (express or implied consent). Fathers were provided resource support in a mailed package in the event they self‐identified as being distressed.

**Table 1:** Characteristics of participants

Characteristic	*N* = 31	%
Marital status		
Married	27	87.1
Common law	4	12.9
Born in Canada		
Yes	18	58.1
No	13	41.9
Ethnicity		
White/Caucasian	22	71.0
Other	9	29.0
Education		
Some elementary/high school	1	3.2
Graduated high school	2	6.5
Some college, trade, or university	4	12.9
Graduated college, trade, or university	20	64.5
Completed graduate school	4	12.9
Language spoken at home		
English	22	71.0
Urdu	1	3.2
Punjabi	1	3.2
Vietnamese	1	3.2
Mandarin	1	3.2
Arabic	1	3.2
Spanish	1	3.2
Farsi	1	3.2
Tagalog	1	3.2
Romanian	1	3.2

### 3.4 Analysis of data

The analysis integrated qualitative and quantitative data through triangulation, complementarity and development (Johnson & Onwuegbuzie, 2004). An interpretive thematic analysis approach permitted identification of statements with similar meanings or ideas (i.e. decontextualizing) (Starks & Brown, 2007). Patterns identified among these statements received reconsideration, given meaning and grouped into themes (i.e. recontextualized) (Starks & Brown, 2007). Transcripts received two independent reviews by two researchers (initial of authors) and a research assistant (initial of author) to ensure confirmability. The central themes and relationships across the narratives and patterns in the statement of the fathers' interviews were arrived through group discussion centred on the research question(s) and Abidin's (1995) and Leigh and Milgrom's (2008) models (i.e. relationships between parenting stress, specifically the parent's and child's characteristics and situational factors).

We used SPSS v19 (IBM SPSS) to analyse quantitative data. Missing data in the PSI and STAI underwent appropriate corrections as per instructions provided in the manuals (Abidin, 1995; Spielberger, 1983). We examined relationships between fathers' parent and child domain stress scores graphically through a scatterplot and statistically through paired *t* test and Pearson correlation. In addition, paired *t* test and Pearson correlation analysed the relationship between the fathers' state or trait anxiety and parent or child domain stress scores. We could not explore the relationships between subscales of parenting stress and PPD, as only one father had depressive symptoms.

## 4 RESULTS

The more detailed contextual information about being a father of a LPI (e.g. thoughts, actions and challenges) is presented first under four themes: ‘Different mindset’, ‘Hard on us’, In the dark—‘Mothers are better informed’ and ‘Not a full, full preemie’. Father's overall stress describes the perceived stressors and levels of stress experienced by father of LPIs and complex nature of their adaption to the birth of their LPIs. Lastly, we present the rate of postpartum depressive symptoms in our sample of fathers of LPIs.

### 4.1 Being a Father of a LPI

#### 4.1.1 "Different mindset"

Fathers described feelings of uncertainty, worry, fear and even denial when they heard that their babies were to be born before the ‘golden cut‐off’ of 37 weeks. Some fathers had forewarnings because of a multiple birth (Father 3) or because of gestational diabetes (Father 2). A premature birth put fathers ‘into a very different mindset’. Father 1 shared a conversation with his wife where they went through a process ‘almost like grieving…I felt we had been robbed of the last month of pregnancy. The pregnancy had come to an end early’. Some fathers tried to make sense of the early birth, with one father taking responsibility ‘I had like bumped her stomach earlier’ and was concerned, but his doctor explained ‘it has nothing to do with it’ (Father 5). Other fathers emphasized frustration in their role as ‘problem‐solvers’ (Father 1). Another ‘went home, grabbed…[a] hospital bag, made sure the playpen was ready and everything for when we brought her home’ (Father 4).

#### 4.1.2 "Hard on us"

Admission of their infants to the NICU alerted, if not alarmed, some fathers. They immediately focused on ‘having mom safe and baby safe’ (Father 5). Father 1 described his feelings: ‘We're a different family group than what you're [NICU] usually set up for but if your policy dictates that [LPIs] come your way, then, you should be ready to deal with it, right’. Another father described his NICU experience as ‘hard for us’ because of guilty feelings. That is, by comparison with others in the NICU, LPIs had a much better prognosis. Consequently, ‘witnessing all the other scenarios with all the babies and seeing the monitors…some babies were in there for like 100 days and you feel for all the other parents’ (Father 5).

#### 4.1.3 In the dark—‘Mothers are better informed’

From their reports, fathers were not privy to all information about their infant. For instance, when asked if his infant had any health problems at birth, father 3 responded, ‘My wife knows more than me. So how about you ask her that’. Another father explained that staff provided information, but ‘I can't remember what exactly it was, but, um, I know they told most of it to [my wife]’ (Father 2).

#### 4.1.4 "Not a full, full preemie"

Hospital providers, regardless of setting (e.g. NICU or normal newborn nursery), encourage normalization of the LPI by counselling fathers that ‘34 weeks is a good time, you know, she's going to be perfectly healthy…96% of babies at 34 weeks do well’ (Father 5). Although fathers understand prematurity, one father, who described his son as ‘not a full, full preemie’, added ‘I'm not a doctor so I'm not even sure what I should be concerned about, if anything anymore’ (Father 1). Another father, whose infant received bottle feeding, took little notice about his infant's uncoordinated suck and swallow reflex, stating, ‘maybe a little bit of milk come[s] out. Like we usually have like a cloth underneath and we do the whole side when we feed her’ (Father 5). All fathers described difficult feeding encounters either for the LPI, parent(s) or both; but in most instances, they do not consider such encounters as problematic or they minimize any difficulty.

### 4.2 Fathers' overall stress

Overall, 31 packages were returned (58% response rate); however, one father did not complete the PSI scale. A couple of fathers (*N* = 4 PSI and *N* = 3 STAI) had missing data which was minimal (<1% for PSI and <7.5% for STAI). Fathers' average age was 33 years (*SD* 4.56, range 28–43 years). Fathers were married (87%), had graduated from a college, trade programme or university (65%), were White/Caucasian (37%), primarily spoke English at home (71%) and were born in Canada (58%). Those born outside Canada (42%) represented several ethnic groups and had resided in Canada for a mean of 9 years (Standard Deviation (*SD*) 5.53). More than half of the total sample (58%) had a combined total household income of >$100,000. Table 1 provides the characteristics of the father.

None of the fathers had an overall raw total stress score equal to and greater than the 85th percentile. They exhibited low life stress as evident from their mean life stress score of 10.0 (*SD* 6.77; range 0–25). Their mean defensive score, however, was 31.4 (*SD* 6.54, range 16–43). Four fathers had clinically statistically significant scores below 24, which suggest that they wanted to minimize the stress in their lives (Abidin, 2012). High scores were evident in subscales of the child domain with one father having a high raw stress score in the child domain (Table 2). High scores were also evident in subscales of the parent domain; to note, the numbers were fewer than in the child domain subscales. Table 3 describes the mean T‐scores for each of the domains and subscales of the PSI to permit comparisons between domains. It is noteworthy that a similar pattern was prominent, where parent domain T‐scores appear to be lower. A positive linear relationship (Pearson's *r* = .566; *p* < .001) between the fathers' child and parent domain stress T‐scores was evident. In other words, if the fathers felt stress because of their children's characteristic(s), they also felt stress related to their own personal parent characteristics. A *t* test revealed a statistically significant difference between the child (mean = 51.30, *SD* 6.16) and parent domain T‐scores (mean = 46.60, *SD* 5.98; see Table 3). That is, child characteristics contribute more to the total stress score than parent characteristics. The following three themes from the fathers' narratives provide contextual information about stressful aspects or lack thereof related to (a) father's sense of competency and role restriction; (b) father–infant interaction; and (c) situational circumstances.

**Table 2:** 85th percentile cut‐off scores for the PSI domains and number of fathers (total *N* = 30) categorized as normal and high stress

PSI^a^ Domain Name	High stress cut‐off score^c^	Normal stress	High stress
		*N*	*N*
Child domain	137	29	1
Distractibility/Hyperactivity	28	24	6
Adaptability	34	26	4
Reinforces parent	16	28	2
Demandingness	28	30	0
Mood	15	25	5
Acceptability	22	29	1
Parent domain	171	30	0
Competence	42	30	0
Isolation	20	29	1
Attachment	20	29	1
Health	15	27	3
Role restriction	24	29	1
Depression	29	30	0
Partner relationship	23	28	2
Total stress	309	30	0
Life stress^b^	27	29	0

a One father did not fill out the PSI.

b One father completed all the domains but did not complete the life stress item at the end of the scale.

c ≥85th percentile.

**Table 3:** Mean and standard deviation for T‐scores in each PSI domain (*N* = 30)

PSI^a^ domain	M	*SD*
Child domain	51.30	6.16
Distractibility/Hyperactivity	54.53	7.84
Adaptability	52.27	7.49
Reinforces parent	49.93	6.44
Demandingness	48.67	6.13
Mood	52.30	10.33
Acceptability	49.37	6.71
Parent domain	46.60	5.98
Competence	45.33	5.77
Isolation	48.03	6.72
Attachment	47.80	6.67
Health	48.60	9.65
Role restriction	47.93	8.05
Depression	46.03	6.08
Partner relationship	47.50	6.55
Total stress	48.57	5.58
Life stress^b^	45.31	13.65

a One father did not fill out the PSI.

b One father completed all the domains but did not complete the life stress item at the end of the scale.

#### 4.2.1 Fathers sense of competency and role restriction

##### Hyper‐vigilance in care

Father 5 described keeping ‘track of everything on a piece of paper…[the] milk we were feeding her, what size of poop she would have, her temperature every feed’. The impulse extended to keeping a rigorous feeding schedule: ‘she feeds every 3 hr continuously. So, they did that in the hospital and then we tried to keep it that way’ (Father 5). For Father 4, the hypervigilance focused on fluid intake: ‘I was, we were waking up, like, at most every 2 hr and getting formula into her, pumping, expressing, just getting food into her’. Gradually, fathers understood the inadvisability of their fastidiousness. Father 1 questioned whether ‘we [should] be so hypervigilant around, um, doing things and that's affected the post birth experienced at home’. Father 2 believed ‘Somebody could have informed us that she would be pretty tired because she wasn't actually ready yet’. He explained, ‘we were watching her all the time, making sure she was still breathing, cause she didn't say much; she didn't squirm around, not like she does now and um, I wasn't expecting her to sleep that much I guess’.

#### 4.2.2 Stressful aspects of father–infant interaction

##### Infant fatigue and parental feeding painful work

One father expressed the sentiments of many. He remembered that he and his wife found themselves pushed to go home, but once home they ‘really wished they [hospital staff] could keep the babies longer…like 10 days. Oh, that would be a big relief’ (Father 3). The father described feeding twins as ‘exhausting’: ‘When I try to feed them, they eat a little bit, they fall asleep, then you put them back and like after 10 min, 20 min they cry again’ (Father 3). Twins (*N* = 6, 8%) were either 36 weeks' gestational age (*N* = 4, 5%) or 34 weeks' gestational age (*N* = 2, 2%). Another father found his infant fatigued differently, depending on the time of the day. He reported, ‘she would maybe take, like say, one third of her feed and then she'd get really tired…But we also found it was times of the day’ (Father 5). Fatigue also appeared to affect breastfed infants. As a father explained, ‘It didn't take her long to get a good latch but she'd, she'd fall asleep a lot’ (Father 4). Father 3 explained that his infant was ‘too weak to suck the milk…Their mouth is too small. Not enough suck’ which necessitated ‘hav[ing] to pump the milk out’. His wife had problems and he described the process as ‘it's a pretty hard job. Yeah, it's a lot of work, painful**’.**

#### 4.2.3 Situational circumstances

##### Unscientific advice from healthcare providers

Fathers appreciated the staff's advice but expressed irritation about inconsistent advice or ‘opinion dressed up as facts’. For instance, Father 1 received ‘four different messages from four different people and everyone conflicting with each other’. Father 3 openly questioned the practicality of the strategies: ‘The nurse teach us some ways, but we don't think they really work’. Frustrated, when even printed materials provided little guidance, he turned to his mother. Another parent (Father 1) recommended that the public health staff should develop a ‘consensus package so you remove that variability of individual advice**’.**

##### Floating

Uncertainty and concern about their infants' health, particularly jaundice and/or weight gain, overshadowed concerns for many fathers. Father 4 stated, ‘[his infant] was literally floating in between…‘okay we need to be cautious’ and ‘okay, we need to go to the hospital.’ Everything about her was just kind of floating the line for the first week’. Father 1 described the management of his infant's jaundice as ‘getting ready for that ride’ and ‘go[ing] through this ten days’ worth of on tenterhooks and having a backpack just in case you were going’. He expressed a sense of relief when he was told his LPI was going to be admitted as ‘It was a case of okay, we're getting our bags; we'll go over to [hospital] and we get the phototherapy; we're done, right?**’**

### 4.3 Postpartum depression in fathers' of late preterm infants

Perinatal clinicians including public health nurses typically adopt a mother and infant centric perspective. One father explained the care as:

very much geared around moms in general…not too many people ask how dad's……there's been times when it's been tough…the only person who's really been asking me how I'm doing is either friends or my wife. (Father 1)

The lack of attention bothered some fathers, given their emotional reactions and need for support. Fathers had a mean state anxiety score of 31.61 (*SD* 6.48, range 23–48). In comparison, fathers had a mean trait anxiety of 33.42 (*SD* 6.48; range 24–50). Although most participants fall in the normal range of anxiety for both scales, one father fell into a high state of anxiety with a score of 48 and another fell into the high trait anxiety diagnosis with a score of 50. We found a relationship between the parent domain and state anxiety, whereby a one‐unit increase in parent domain stress was associated with a mean increase in state anxiety of .11 units (*p* = .039). In this study, only one (3%) father had depressive symptoms. Consequently, we could not explore the relationships between subscales of parenting stress and PPD.

## 5 DISCUSSION

Fathers experienced challenges in adapting to the birth of the LPIs, likely because the untimely transition to fatherhood put them in ‘different mindset’. In those instances where the infant was in the high‐tech NICU, with highly compromised infants, fathers experienced moral distress given the better prognosis of their infant. The fathers' narratives, however, suggest that they normalized the infants' special needs with emerging assertions being hospital staff not emphasizing their infant's physiological and metabolic immaturity, or it was a strategy to manage anxiety and uncertainty as evident from hypervigilance in care of their infant (Wong et al., 2016). The hypervigilance employed by fathers suggests they can cope with anxiety and uncertainty of caring for their infants (Wong et al., 2016), as explained by their low parental stress scores. Social comparison where mothers evaluated their preterm infant in relation to another premature infant enabled them to cope with the stressful circumstances of having a premature infant (Blanchard, Blalock, DeVellis, DeVellis, & Johnson, 1999). For fathers in our study, however, this social comparison orientation of comparing their LPI to other premature infants elicited guilt and made it more difficult for them to adapt to the birth of their LPI.

First time fathers of LPIs felt excluded or on the periphery (excluded or in the shadows) because mothers remain the primary focus of attention (Benzies & Magill‐Evans, 2015). In our study, fathers also felt excluded for the same reasons. Ironically, this omission could explain their low stress levels. They would lack the knowledge to appreciate the extent of the challenges associated with caring for LPIs. About 13% of fathers had defensive responding scores below (Goodman, 2004), which implie that the low stress scores likely do not reflect the level of fathers' stress (Abidin, 2012).

Fathers in this study were married, had relatively high levels of education and had low life stress as evident from their mean life stress score of 10.0 (*SD* 6.77; range 0–25). Experiences in life, particularly early positive life experiences, can improve an individual's ability to respond to and cope with adversity given changes in brain circuitry and function, making them resilient to anxiety and depression (McEwen, Gray, & Nasca, 2015). New circumstances can, however, challenge the individual's capacity given the plasticity of the brain throughout life (McEwen et al., 2015). Anxiety and uncertainty are typical responses for expectant and new parents (Svensson, Barclay, & Cooke, 2006), and the cumulative effects of an untimely transition to parenthood combined with stressful aspects of LPI interaction (i.e. infant fatigue and parental feeding painful work) and situational stressors (i.e. unscientific advice from healthcare providers, floating between having their infant home or being rehospitalized) could explain the stress and anxiety felt by some fathers (McEwen, 2006). Moreover, the LPIs' characteristics become problematic when they have a direct impact on the father (Wong et al., 2016).

Animal and human studies reveal adaptive changes in the brain and hormonal responses (e.g. testosterone, cortisol and prolactin) intended to promote closeness to mother through nurturing and supportive behaviours and prepare for parenthood (Berg & Wynne‐Edwards, 2001; Edelstein et al., 2015, 2017; Wynne‐Edwards, 2001). Stress and anxiety can hinder these adaptive changes and have an impact on social behaviour in ways that hinders father's ability to provide social support and interact with his LPI in ways promotes cognitive, social and emotional development of the infant (Berg & Wynne‐Edwards, 2001; Carré, McCormick, & Hariri, 2011; Edelstein et al., 2015, 2017; Wynne‐Edwards, 2001). Fathers seem beset by the bio‐psycho‐social demands of their infants, as exhibited by the stress in the child and parent domains and anxiety. Although only one father's total child domain score was classified as high, more fathers had high stress scores in child and parent domain subscales. In our sample, the child domain contributed statistically significantly to the total stress scores when compared with the parent domain. Fathers' narrative illuminates the sources of stress related to feeding experiences, which were prolonged, exhausting and painful, requiring heightened attentiveness about feeding and jaundice, created uncertainty related to rehospitalization. The child domain contributed statistically significantly to the total stress score on the PSI. With few exceptions, fathers had low STAI anxiety scores. An association appeared between parent domain and state anxiety. The grief or loss of normal pregnancy and the NICU experience could also contribute to the fathers' anxiety. We did not examine the impact of paternal stress, anxiety and depression on maternal perinatal mental health.

In view of the theoretical models guiding our study, we anticipated that at 6–8 weeks following birth of their LPIs some fathers would manifest depressive symptoms. However, this did not occur with our sample. Only one father self‐reported depressive symptoms. The literature examining PPD in fathers of LPIs is sparse; one study (Mehler et al., 2014) reported a PPD rate (EPDS score >9) of 13% between days 2 and 10 days following birth of the LPIs with a marked reduction (50%) at term. The same study reported increased risk of PPD among fathers of LPIs compared with fathers of full‐term infants. A meta‐analysis of postpartum depression in fathers reported statistically significant variability in rates of PPD with rates being lowest in the first 3‐month postpartum and highest in the 3‐ to 6‐month postpartum period (Paulson & Bazemore, 2010). For certain, our small sample impedes precision around the estimate of rate of PPD among fathers of LPIs. A larger number of participating fathers and repeated assessments of PPD would enhance our understanding of paternal perinatal mental health. Future studies should compare stress including child and parent domains, anxiety and depression over time between fathers' of LPIs and term infants.

## 6 LIMITATIONS

We wish to draw the reader's attention to some limitations in our study. The research team first contacted mothers to identify potential participants with mothers reporting that 113 eligible fathers (68%) refused to participate. Maternal gatekeeping negatively influences father involvement (Fagan & Barnett, 2003); the resulting selection bias has an impact on reliability or accuracy of study findings and external validity. Consequently, we recommend that future studies should directly approach fathers in the recruitment process to reduce selection bias. Most fathers enrolled in this study were married, had relatively high levels of education and could read, write and speak English fluently. This sample limits both transferability and generalizability of findings to fathers with different demographic characteristics (e.g. low‐income). The chosen instruments could lack sensitivity to identify fathers with high stress (overall, child and father domains) and anxiety as we applied normative data which do not account for standardized differences in tests between American and Canadian populations (Harrison, Armstrong, Harrison, Lange, & Iverson, 2014; Williamson & Williamson, 1989). Lastly, the study interviewed a small sample of fathers (*N = *5). Despite the limited number of fathers that consented to be interviewed, the qualitative data appeared to have sufficient ‘information power’ given the established theory guiding our data collection and level of father's engagement during the interview (i.e. quality of dialogue), and the analytic strategy of triangulation (Malterud, Siersman, & Guassora, 2016). The small sample, besides the limited variation in participants, challenges the adequacy (i.e. sufficiency and quality) of qualitative data and transferability of the findings (Malterud et al., 2016).

## 7 CONCLUSION

In summary, the hospital system apparently leaves fathers of LPIs unprepared to participate in their care. Mixed messages from clinicians add to the problem. The fragility of LPIs further compounds paternal anxieties and stress. The situation can interfere with how much support fathers can provide mothers and ultimately to health outcomes of their LPIs. The ethos of care, both in the hospital and community, should treat fathers more equitably. Future studies should compare the influence of the child domain on stress between fathers' of LPIs and full‐term infants and examine the mediating effect of life stress on this relationship. A full‐scale mixed‐methods study would enhance our understanding of what it does mean to be a father of a LPI, especially the normalization of care and factors contributing to this, as normalization may block developmentally sensitive parenting (i.e. normalization of LPI) (Wong et al., 2016).

## CONFLICT OF INTEREST

None declared.

## AUTHOR CONTRIBUTIONS

SSP, SR, GC, AD, AKL and MY conceptualized, planned and implemented the study. SSP, SR, LMO, MC, and TW completed the analysis with input from CG, AD, and MY. SSP drafted the article and all authors critically examined, revised and approved the final version.

## ETHICAL APPROVAL

The Conjoint Health Research Ethics Board at the University of Calgary (Ethics ID: E‐25040) and Department Heads within Alberta Health Services provided approvals.
